# TIAM2S Operates Multifaced Talents to Alleviate Radiosensitivity, Restrict Apoptosis, Provoke Cell Propagation, and Escalate Cell Migration for Aggravating Radioresistance-Intensified Cervical Cancer Progression

**DOI:** 10.3390/cells14050339

**Published:** 2025-02-26

**Authors:** Pei-Chin Chuang, Wen-Hong Su, Ching-Hua Hsieh, Eng-Yen Huang

**Affiliations:** 1 Department of Medical Research, Kaohsiung Chang Gung Memorial Hospital, Kaohsiung 833401, Taiwan; stemcorelab@cgmh.org.tw (P.-C.C.); whsu0909@gmail.com (W.-H.S.); 2 Department of Biotechnology, Kaohsiung Medical University, Kaohsiung 807017, Taiwan; 3 Department of Obstetrics and Gynecology, Kaohsiung Chang Gung Memorial Hospital, Chang Gung University College of Medicine, Kaohsiung 833401, Taiwan; 4 Department of Plastic Surgery, Kaohsiung Chang Gung Memorial Hospital, Chang Gung University College of Medicine, Kaohsiung 833401, Taiwan; 5 Department of Radiation Oncology, Kaohsiung Chang Gung Memorial Hospital, Chang Gung University College of Medicine, Kaohsiung 833401, Taiwan; 6 School of Traditional Chinese Medicine, Chang Gung University, Taoyuan 333323, Taiwan

**Keywords:** TIAM2S, radioresistance, apoptosis, cell propagation, cell migration, cervical cancer progression

## Abstract

Radioresistance remains a major obstacle in cervical cancer treatment, frequently engendering tumor relapse and metastasis. However, the details of its mechanism of action remain largely enigmatic. This study delineates the prospective impacts of short-form human T-cell lymphoma invasion and metastasis 2 (TIAM2S) involving the radiation resistance of cervical cancer. In this study, we established three pairs of radioresistant (RR) cervical cancer cells (HeLa, C33A and CaSki) and their parental wild-type (WT) cells. We revealed a consistent augmentation of *TIAM2S*, but not long-form human T-cell lymphoma invasion and metastasis 2 (*TIAM2L*) were displayed in RR cells that underwent a 6 Gy radiation administration. Remarkably, RR cells exhibited decreased radiosensitivity and abridged apoptosis, as estimated through a clonogenic survival curve assay and Annexin V/Propidium Iodide apoptosis assay, respectively. TIAM2S suppression increased radiosensitivity and enhanced cell apoptosis in RR cells, whereas its forced introduction modestly abolished radiosensitivity and diminished WT cell apoptosis. Furthermore, TIAM2S overexpression notably aggravated RR cell migration, whereas its blockage reduced WT cell mobilities, as confirmed by an in vitro time-lapse recording assay. Notably, augmented lung localization was revealed after a tail-vein injection of CaSki-RR cells using the in vivo short-term lung locomotion *BALB/c* nude mouse model. TIAM2S impediment notably reduced radioresistance-increased lung locomotion. This study provides evidence that TIAM2S may operate as an innovative signature in cervical cancer that is resistant to radiotherapy. It displays multi-faceted roles including radioprotection, restricting apoptosis, promoting cell proliferation, and escalating cell migration/metastasis. Targeting TIAM2S, together with conventional radiotherapy, may be an innovative strategy for intensifying radiosensitivity and protecting against subsequent uncontrolled tumor growth and metastasis in cervical cancer treatment.

## 1. Introduction

Cervical cancer is the fourth most prevalent cancer worldwide; current treatment options include surgery, radiotherapy, chemotherapy, and immunotherapy [1,2]. Though relatively controlled for several decades in high-income countries, cervical cancer remains the main cause of cancer-related deaths among women in low- and lower-middle-income countries [3]. Radiotherapy is the major radical treatment option for patients in stages IB3 and IIA2–IVA, and an optional radical therapy for those in stages IB1–2 and IIA [2]. However, after radiotherapy, recurrence occurs within two years among 20–40% of patients, with local and distant metastasis being the main patterns, often leading to death [4,5,6]. Radioresistance is a main cause of radiotherapy failure among patients with cervical cancer, and those with recurrent cancer frequently have poor prognosis with a 5-year overall survival of around 10–20% [7,8]. Therefore, developing a better understanding of radioresistance in cervical cancer is a hot spot for radio-oncological research.

Human T-cell lymphoma invasion and metastasis 2 (TIAM2) belongs to the TIAM family. The human gene *TIAM2* is located on chromosome 6q25.2, and is the homolog of human *TIAM1* [9]. TIAM1 is a Rac-specific guanine nucleotide exchange factor first identified from gene screening for increased invasiveness of T lymphoma cell lines [9]. Human *TIAM1* is well defined as a crucial regulator of neurotrophic factors and ephrin B ligand-mediated neuronal functions, including neurite outgrowth modulation, dendritic spine development, and neuron polarization and migration [10,11]. Human *TIAM2* encodes two mRNA transcripts: the long transcript (NM_012454.3; *TIAM2 varian1*) encodes *TIAM2L*, a protein comprising 1701 amino acid residues, and the short transcript (NM_001010927.2; *TIAM2 varian2*) encodes TIAM2S, a 626-residue protein. Notably, compared to studies of TIAM1, details regarding the biological impact of TIAM2S are still limited. It has been reported that TIAM2S is enriched in both fetal and adult brains, participating in the modulation of neurite outgrowth. Hence, TIAM2S-positive microglia have been demonstrated to enhance inflammation and neurotoxicity [12] through soluble ICAM-1-mediated immune priming [13]. Chu CH et al. further demonstrated that TIAM2S overexpression may be a critical regulator for the hippocampal–medial prefrontal cortex network in progressing age-related spatial memory impairment [14]. Recently, an animal study demonstrated that TIAM2S aggravates a proinflammatory immune microenvironment that enhances colorectal carcinogenesis, implying that abnormal TIAM2S expression probably contributes to human cancer malignancies. Interestingly, by using Gene Set Enrichment Analysis to analyze the Gene Expression Omnibus Accession Viewer online public clinical database, we revealed that T-cell lymphoma invasion and metastasis 2 (TIAM2) increased in the group of the cervical cancer patients experiencing intrinsic radioresistance after 6 months, in comparison with those who saw complete response in the same timeframe (Supplementary Figure S1). However, whether TIAM2S participates in regulating radioresistance progression in cervical cancer remains largely unknown. This study was undertaken to delineate the biological impacts of TIAM2S in regulating multiple radiation-resistance processes, including radiosensitivity post radiation administration, cell growth/propagation, radioresistance-associated cell apoptosis, and subsequent cell migration/metastasis in cervical cancer.

## 2. Materials and Methods

### 2.1. Experimental Animals

Female *BALB/c* nude mice (6–8-weeks old) were obtained from the National Animal Center, Taiwan. In this study, mice were housed in the animal center and light was provided between 6:00 a.m. and 6:00 p.m. Mice were maintained on a standard diet of chow and water ad libitum. All the procedures and protocols were approved by the Institutional Animal Care and Use Committee of Chang Gung Memorial Hospital. (Approval code: CGMH, 2021120805; valid period: 1 August 2023–31 July 2026).

### 2.2. Radioresistant Cervical Cancer Cells

Human cervical carcinoma cells (HeLa, CaSki and C33A) were obtained from the American Type Culture Collection (ATCC, Manassas, VA, USA). The culture procedure and media used were chosen according to the same directory. Briefly, sub-confluent cells were cultured at 37 °C under an atmosphere of 5% CO_2_ in a 25T flask and subjected to an irradiation administration procedure described elsewhere [15]. To establish radioresistant cervical cancer cell clones, cells were plated, cultured, and subjected to 6 Gy radiation administration at a rate of 4 Gy per minute using a linear accelerator (Varian Medical Systems, Palo Alto, CA, USA). In a parallel experiment, there was another set of non-irradiated flasks (HeLa, C33A and CaSki cells), and this was defined as the parental wild-type group. The culture media in each flask was replaced after 24 h of cultivation to remove detached cells. Cells resistant to 6 Gy radiation administration were proliferated in the same cultivated media, which was replaced every 3 days. All of the vital and stable radioresistant colonies were expanded in vitro for over six months to confirm the radioresistant phenotype prior to the start of the study. Additionally, parental wild-type clones for each cell type (HeLa, C33A and CaSki) were established under identical procedures without irradiation.

### 2.3. Reverse Transcription (RT) and Quantitative Polymerase Chain Reaction (QPCR)

The procedures for total ribonucleic acid (RNA) extraction, nucleotide quality determination, concentration estimation, reverse transcription and quantitative PCR have been described elsewhere [16]. Total RNA was extracted from the cells, mixed with a RT-Mixture reagent (Ambion, Inc., Austin, TX, USA) and then subjected to reverse transcription in complementary deoxynucleic acid (cDNA). Later, the cDNA templates were mixed with the PCR mixtures using the 2× TaqMan^®^ Universal PCR Master Mix. PCR amplification was executed using the ABI 7900 Detection System (Applied Biosystems, Foster City, CA, USA) according to the manufacturer’s instructions. The *TIAM2S* transcript level was estimated by PCR to generate 170-bp amplicons (accession number: NM_001010927.2; forward primer 5′-CAGGTTTCTTTGAACTTTTCC-3′, reverse primer 5′- GGAGGCGGTCTGCATCAG-3′), and the expression of *TIAM2L* transcripts was estimated following the same method to generate the 131-bp amplicons (accession number: NM_012454.3; forward primer 5′-CAGGATGCGACGGATCAGT-3′, reverse primer 5′-CCTGCACATCTGTATTCAGTCCAT-3′).

### 2.4. siRNA and Plasmid Transfection

siRNA transfection was executed as described elsewhere [17]. Briefly, *siRNAs against human TIAM2S* (sense 5′-ACAGCCGACUUAGAUGUGAUCCGTT-3′; antisense 5′-AACGGAUCACAUCUAAGUCGGCUGUUG-3′), and corresponding guanine/cytosine content-matched *scrambled siRNAs* (negative control; sense 5′-UUCUCCGAACGUGUCACGUTT-3′; antisense 5′-ACGUGACACGUUCGGAGAATT-3′) (Thermo fisher scientific, Carlsbad, CA, USA), were individually transfected into cells at a final concentration of 10 or 30 nM. Oligonucleotides were delivered to the cells using Lipofectamine 2000 (Thermo fisher scientific, Carlsbad, CA, USA). The pcDNA3.1-Myc-His A tagged Human TIAM2S plasmid was constructed [18] and kindly provided by Prof. H. Sunny Sun’s laboratory, Institute of Molecular Medicine, National Cheng Kung University Medical College, Taiwan.

### 2.5. Clonogenic Survival Curve Assay After Irradiation Treatment

A clonogenic assay was performed as described elsewhere [15]. Cells were seeded, cultivated in 25 T flasks, and then subjected to different treatment conditions (pre-transfection with pcDNA3.1-Myc-His A-TIAM2S or vector alone, *siRNA of scrambled NC* or *siRNA of TIAM2S*, respectively). After 72 h of transfection, cells in 25 T flasks were irradiated (0, 2, 4, 6, and 8 Gy, using a linear accelerator). After irradiation, the cells were trypsinized, harvested, counted (100–10,000/well, according to the radiation dose) and immediately plated in six-well plates (plating was performed immediately after irradiation). After 14 days following irradiation, cell colonies were fixed and stained using glutaraldehyde (6.0% *v*/*v*) and crystal violet (0.5% *w*/*v*), respectively. A clonogenic surviving colony was considered when 50 or more cells were counted. Normalization to 0 Gy in each condition was established according to plating efficiency (PE) (Table S1). The surviving fraction equaled the number of colonies/(number of cells plated × PE). The survival curve was drawn according to the survival rate (log) and dose.

### 2.6. Flow Cytometric Analysis of Annexin V/Propidium Iodide Apoptosis Assay

To measure cell apoptosis rate, the Annexin V/Propidium Iodide apoptosis assay was executed according to the manufacturer’s instructions [19]. In brief, the designed CaSki and C33A cells were incubated with fluorescein isothiocyanate (FITC) Annexin V and a buffer containing propidium iodide; then, they were assessed by LSR II flow cytometry (BD Biosciences, Franklin Lakes, NJ, USA). The Annexin V-positive cells (sum of the annexin V-positive/PI-negative as well as the annexin V-positive/PI-positive cells) were described as apoptotic.

### 2.7. BrdU Incorporation Assay

To estimate cell proliferation, the 5′-bromo-2′-deoxyuridine (BrdU) cell proliferation assay—a non-isotopic immunoassay for the in vitro quantification of BrdU incorporation into the newly synthesized DNA of actively proliferating cells—was executed according to the manufacturer’s protocol. Briefly, serum-starved cells were cultivated and then subjected to the cell BrdU proliferation assay. Six hours before harvest, BrdU (100 µg/mL) was added into the culture media, and cells were fixed and stained with an anti-BrdU antibody using commercial kits (cell proliferation assay kit purchased from Amersham Pharmacia Biotech, Little Chalfont, UK). BrdU-positive cells (newly proliferating cells) were shown by red fluorescence; for the nuclear counterstain, DAPI (4′,6-diamidino-2-phenylindole) staining showed blue fluorescence. Nine to twelve randomly selected fields were inspected for counting BrdU-positive cells, and at least 500 cells were counted in each treatment group.

### 2.8. Time-Lapse Recording of Cell Migration

To measure the cell migrative rate, a time-lapse recording of a cell migration assay was executed according to the manufacturer’s instructions [15]. Briefly, designated cells were plated and recordings were taken hourly using a microscope equipped with a camera system (Axiovert 200; Carl Zeiss Micro Imaging GmbH, Welwyn Garden City, UK). The acquired images were subjected to semi-automatic recording after every single cell movement (in µm) using AxioVision Rel. version 4.8 software, and the cell migrative speed was defined as the single cell movement (in µm)/8 h.

### 2.9. Fluorescent Labeling of the Cervical Cancer Cells

Designed cells (1 × 10^7^ cells/mL) were plated and incubated with a reduced-serum Opti-minimal essential medium (MEM) containing fluorescent CellTracker™ Green CMFDA (5-chloromethylfluorescein diacetate) (Thermo fisher scientific, Carlsbad, CA, USA) or CellTracker™ Orange CMRA Dye (10 μmol/L) for thirty minutes at 37 °C, according to the manufacturer’s instructions [20]. Cells were washed and incubated with dye-free phosphate-buffered saline (PBS) for another thirty minutes. Then, the harvested CaSki or C33A cells were immediately injected into *BALB/c* nude mice.

### 2.10. In Vivo Short-Term Locomotion of Tumor Cells to Lungs in Nude Mice Model

To measure lung metastasis, as described elsewhere [20,21], the designed wild-type and radioresistant CaSki and C33A cells were labeled with fluorescent CellTracker™ Green CMFDA or CellTracker™ Orange CMRA Dye (10 μmol/L), and then mixed WT and RR cells at a ratio of 1:1. A total amount of 2 × 10^6^ cells were injected into each mouse by tail-vein injection. Lungs were harvested after 0.5 or 24 h of injection, and sectioned at 10 mm in a cryostat to estimate the locomotion of fluorescent dye-stained tumor cells to lungs. The green or red fluorescent cells in lungs were counted under fluorescence microscopy (AxioVision; Carl Zeiss, Welwyn Garden City, UK).

### 2.11. Western Blot

Cell extract was lysed in RIPA buffer (50 mM Tris-HCl, pH 7.4, 150 mM NaCl, 1% NP-40, 1M Dithiothreitol, 100 mM PMSF Protease Inhibitor, 0.5% sodium deoxycholate, 0.1% SDS, 1% Triton X-100), homogenized, centrifuged, and then the supernatant was harvested. Total cell lysate (approximately 100 μg) was loaded onto 8% SDS-PAGE, and electro-transferred to polyvinylidene difluoride membranes using semi-dry transfer. The membranes were treated with methanol and blocked for 1 h at room temperature with 5% bovine serum albumin in TBST (10 mM Tris-HCl, pH8.0, 150 mM NaCl, and 0.05% Tween 20). The membranes were probed with the primary antibodies including anti-TIAM2 (Abcam, Cambridge, UK), and anti-Myc tag antibodies (Cell Signaling Technology, Danvers, MA, USA). Anti-β-actin (Sigma-Aldrich, Saint Louis, MO, USA) was used as the loading control. The membrane was then washed with PBS Tween-20 Buffer (PBST) for 1 h and detected using the enhanced chemiluminescence (ECL) detection system (NEL105001EA, Western Lightning Plus-ECL, PerkinElmer, Hopkinton, MA, USA).

### 2.12. Statistical Analysis

Each set of experimental data was analyzed using one-way analysis of variance (ANOVA) and module analysis of variance using Prism software version 4.02 (GraphPad Software, San Diego, CA, USA); data are shown as mean ± standard deviation (SD). The log rank test was used to estimate differences between groups of Kaplan–Meier survival curves. Student’s *t*-test was applied to compare two samples. The Mann–Whitney U Test, a non-parametric statistical test, was used to compare two samples or groups to determine if there were statistically significant differences between them. Tukey’s test was applied to measure whether the differences between the experimental results of the paired groups were significant, while Dunnett’s test was utilized to individually compare the results among three groups when the F-test found significance.

## 3. Results

### 3.1. Amplified TIAM2S Level in Established Radioresistant HeLa, CaSli, and C33A Cell Clones

**Figure 1:**
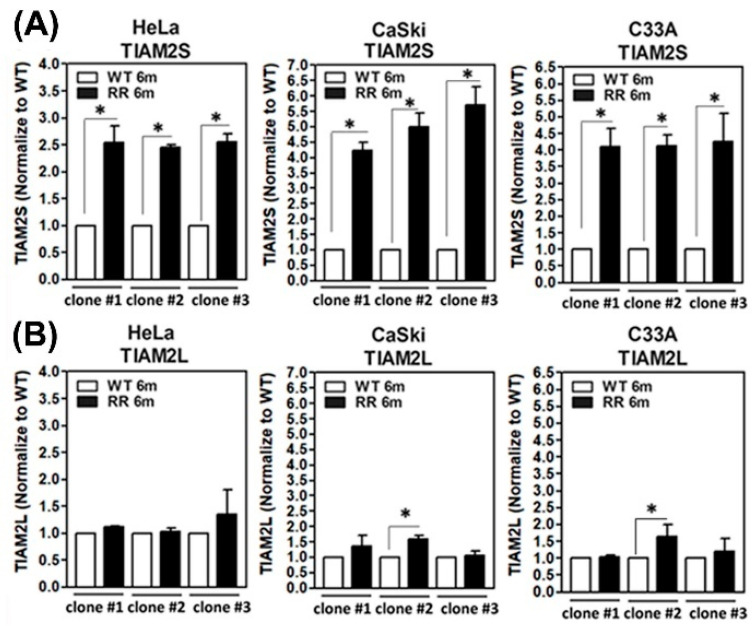
Augmented *TIAM2S* but not *TIAM2L* shown in radioresistant (RR: 6 months) clones compared to their parental wild-type (WT) clones generated from HeLa, CaSki and C33A cervical cancer cells. Expression levels of (**A**) *TIAM2S* and (**B**) *TIAM2L* in WT and RR cervical cancer cells: HeLa, CaSki, and C33A cells. Cells were cultivated and collected, the total RNA was extracted, and expression levels of *TIAM2S* and *TIAM2L* transcripts were measured by quantitative RT-PCR analysis, as described in Section 2.3. The results were from three independent clones of HeLa, CaSki, and C33A cells; each clone was repeated at least three times, and the results were similar. Data are shown as means ± SD. Asterisk symbol (*) denoted significant differences between groups when Mann–Whitney U Test analysis yielded *p* < 0.05.

We have previously established three radioresistant cervical cancer cell lines [15]. The detailed procedure for generating radioresistant (RR) clones is described in Section 2.2. Three clones from HeLa, CaSki and C33A cervical cancer cells were established. The parental wild-type (WT) cell clones were generated under identical procedures without irradiation. All of the vital RR colonies after 6 Gy irradiation administration were expanded in vitro for over 180 days to confirm the radioresistant phenotype. Human *TIAM2* is located on chromosome 6q25.2 and encodes two transcripts, designated as *TIAM2L* (*TIAM2 varian-1*, NM_012454.3) and *TIAM2S* (*TIAM2 varian-2*, NM_001010927.2) for the long and short forms, respectively. We then assessed the transcript levels of two members of the TIAM2 family (*TIAM2S* and *TIAM2L*) in our established human parental WT and RR cervical cancer cell clones of HeLa, CaSki, and C33A, as described in Section 2.3. We found a notable and consistent increase in *TIAM2S* transcript in three radioresistant groups compared to those from their parental wild-type group (Figure 1A). This phenomenon was constant among the various clones of the three cell lines. Although some of the clones showed minor induction of *TIAM2L* in RR groups compared to those in the WT group, nonetheless yet shown in a consistent manner (Figure 1B). We thus honed our focus to explore the role of TIAM2S in radioresistance-associated cervical cancer progression.

### 3.2. TIAM2S Manifested as a Radioprotector and Exhibited a Crucial Role in Modulating the Radiosensitivity of Radioresistant CaSki and C33A Cervical Cancer Cells

**Figure 2:**
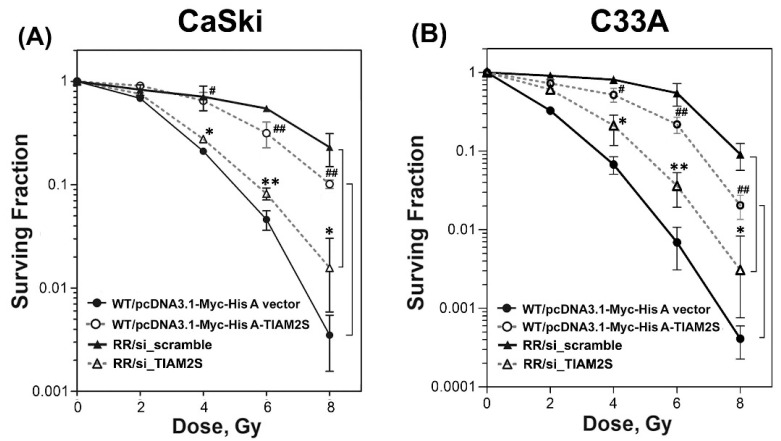
Radiation survival curve of cervical cancer cells was executed on cells in log-phase growth to estimate radiosensitivity. Wild-type (WT) cells were transfected with pcDNA3.1-Myc-His A vector alone (3 μg) or pcDNA3.1-Myc-His A-TIAM2S (3 μg), whereas radioresistant cells were transfected with *siRNA of scrambled NC* (30 nM, also termed si_scramble) or *siRNA of TIAM2S* (30 nM; also termed si_TIAM2S), respectively, as described in Section 2.4. CaSki and C33A cells were plated, irradiated, and analyzed to estimate the clonogenic surviving fraction at the designated dosages of radiation. All of the clonogenic assays were repeated at least three times, as described in Section 2.5. The survival curve showed decreased radiosensitivity in the radioresistant clones of (**A**) CaSki and (**B**) C33A cells compared to their parental wild-type cells. RR cells transfected with *siRNA of TIAM2S* abridged the surviving fraction (elevated radiosensitivity), while WT cells transfected with pcDNA3.1-Myc-His A-TIAM2S increased it (decreased radiosensitivity). Data are shown as means ± SD. Solid filled circles curve: WT-pcDNA3.1-Myc-His A vector alone; dotted open circles curve: WT- pcDNA3.1-Myc-His A-TIAM2S; solid filled triangle curve: RR-siRNA of scrambled negative control; dotted open triangle curve: RR-siRNA of TIAM2S. Each experiment was performed in triplicate, and the results were similar. * *p* < 0.05, ** *p* < 0.01, for the si_TIAM2S group vs. the si_scramble group. # *p* < 0.05, ## *p* < 0.01, for the pcDNA3.1-Myc-His A-TIAM2S group vs. pcDNA3.1-Myc-His A vector group.

Next, we investigated the impact of TIAM2S in regulating radiosensitivity after irradiation administration. First of all, we re-challenged the established stable radioresistant clones with various dosages of radiation (0, 2, 4, 6, and 8 Gy) and executed a clonogenic survival curve assay to estimate radiosensitivity. As shown in Figure 2A,B, we found that all of the RR clones (CaSki and C33A) consistently displayed elevated survival fractions compared to those of parental wild-type (WT) clones after 6 Gy radiation treatments. These data further confirmed that the surviving populations we selected and defined as stable RR clones of CaSki and C33A cells were, indeed, radioresistant. Further, we then explored the influence of gain or loss of TIAM2S function on radiosensitivity among WT and RR cell clones. Radioresistant CaSki and C33A cell clones (with higher TIAM2S expression) were transfected with the *scramble siRNA* (RR/si_scramble group, a negative control for siRNA) or transfected with the *siRNA of TIAM2S* (RR/si_TIAM2S group) for 72 h, respectively. In contrast, parental CaSki-WT cells (with lower or little TIAM2S expression) were transfected with the pcDNA3.1-Myc-His A vector alone (WT/pcDNA3.1-Myc-His A vector group) or transfected with pcDNA3.1-Myc-His A-TIAM2S construct (WT/pcDNA3.1-Myc-His A-TIAM2S group) for 72 h, respectively, as described in Section 2.4. Next, cells were subjected to different dosages of radiation administration. After 10 to 14 days, surviving colonies were fixed, stained, and counted, as described in Section 2.5. Forced TIAM2S introduction into CaSki-WT cells significantly enhanced the clonogenic survival rate compared to the CaSki-RR cells transfected with the empty vector alone after 6 Gy radiation treatments (Figure 2A). In contrast, in CaSki-RR cells, TIAM2S suppression notably enhanced the rate of surviving populations compared to the CaSki-RR group, which was transfected with scrambled negative controls. A similar result was revealed in C33A (Figure 2B). Our data suggest that TIAM2S may display an innovative anti-radioresistance molecule and play a crucial role in modulating radiosensitivity. 

### 3.3. TIAM2S Played an Anti-Apoptosis Role in Cervical Cancer Cells

**Figure 3:**
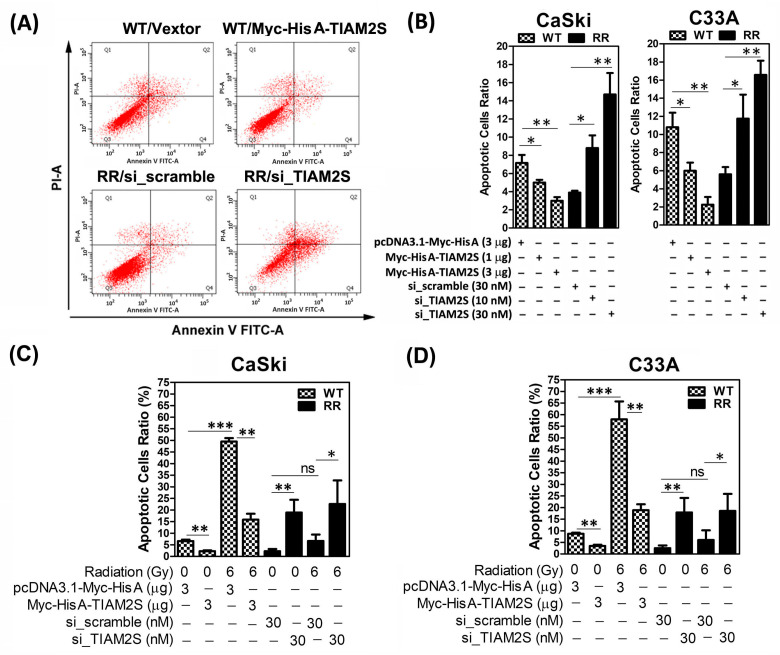
Decreased apoptotic cell ratio was shown in radioresistant CaSki and C33A cells, and knockdown of TIAM2S reinforced apoptosis in radioresistant cervical cancer cells. (**A**) Representative figure of flow cytometric analysis of Annexin-V/PI double staining shown on CaSki-WT and-RR cells. Designed cells were pre-transfected with pcDNA3.1-Myc-His A-TIAM2S (3 μg) or vector alone (3 μg), *siRNA of scrambled NC* (30 nM) or *siRNA of TIAM2S* (30 nM), respectively. After 72 h of transfection, cells were collected and the apoptosis rate was assessed, as described in Section 2.6. A summary of the apoptotic cell ratio (sum of annexin V-positive/PI-negative and annexin V-positive/PI-negative cells) is shown in (**B**). We investigated the in vitro relevance of TIAM2S involved in cell apoptosis regulation under with or without 6 Gy radiation treatment. (**C**) CaSki-WT or (**D**) C33A-WT cells were pre-transfected with pcDNA3.1-Myc-His A-TIAM2S construct or vector alone, respectively, whereas CaSki-RR cells were pre-transfected with *siRNA of scrambled NC* (30 nM) or *siRNA of TIAM2S* (30 nM), respectively. After 72 h of transfection, designed cells were resubjected to 0 Gy (without radiation treatment) or 6 Gy radiation treatment. Apoptotic cell ratio was assessed at 48 h after 0 Gy or 6 Gy radiation treatment, and a summary is provided. Data were obtained from three independent experiments in triplicate and are shown as the mean ± SD. * *p* < 0.05, ** *p* < 0.01, *** *p* < 0.001; “ns” indicates no significance (*p* > 0.05).

Hence, to elucidate whether the elevated survival rate of radioresistant CaSki and C33A was due to cell apoptosis suppression, a flow cytometric analysis of FITC Annexin V staining was carried out, as described in Section 2.6. Briefly, to quantify the rate of apoptosis, apoptotic cells were identified using the Annexin-V/FITC kit (Biouniquer, Franklin Lakes, NJ, USA), following the manufacturer’s protocol. Figure 3A,B show that the basal apoptotic ratio of the CaSki-WT control group was higher than the CaSki-RR scramble control group. The ectopic overexpression of TIAM2S was executed by transfection of various dosages (1 and 3 μg) of the pcDNA3.1-Myc-His A-TIAM2S construct into CaSki-WT cells for 72 h, while TIAM2S levels were decreased by the transfection of *siRNA of TIAM2S* (10 and 30 nM) into CaSki-RR cells for 72 h, respectively. TIAM2S overexpression markedly reduced the apoptotic cell ratio of CaSki-WT cells in a dose-dependent manner, while a TIAM2S level decrease dose-dependently increased apoptotic cell populations in CaSki-RR cells (Figure 3A,B). We then further investigated the in vitro impact of TIAM2S in regulating cell apoptosis under re-challenge with or without 6 Gy radiation treatment. As shown in Figure 3C, CaSki-WT cells were pre-transfected with 3 μg of pcDNA3.1-Myc-His A-TIAM2S construct for 72 h, before being subjected to 6 Gy radiation treatment. After 48 h following 6 Gy radiation treatment, radiation notably induced the apoptotic rate and the ectopic overexpression of TIAM2S effectively abolished radiation-induced cell apoptosis in CaSki-WT cells. Notably, TIAM2S expression inhibition markedly induced CaSki-RR cell apoptosis both in 0 Gy (without radiation) and 6 Gy radiation treatment groups. Six Gy radiation treatment had no further significant influence on the apoptotic rate in CaSki-RR cells compared to those in the 0 Gy treatment group. The net increase in apoptosis induced by radiation in CaSki cells without TIAM2S knockdown was comparable to that in CaSki-RR cells with TIAM2S knockdown. A similar phenomenon was revealed in C33A (Figure 3D). Our data suggest that TIAM2S may act as an anti-apoptosis factor to aid cells in overcoming apoptosis of CaSki-WT and C33A-WT cells. Hence, although TIAM2S knockdown significantly enhances radiosensitivity, our data suggest that its radio-sensitizing effect was not related to apoptosis in CaSki-RR and C33A-RR cells.

### 3.4. TIAM2S Exhibited Pro-Cell Propagation, While TIAM2S Blockage Effectively Abridged CaSki and C33A Radioresistant Cell Propagation

**Figure 4:**
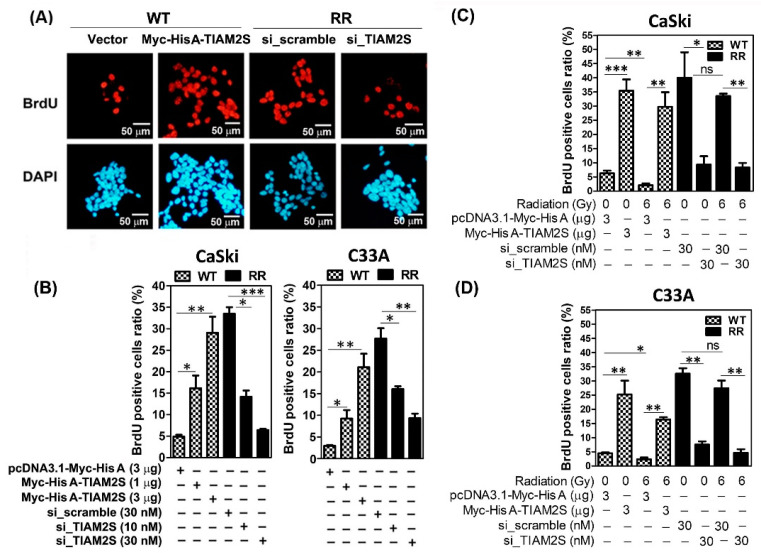
Augmented cell proliferation was shown in radioresistant CaSki and C33A cells in vitro and TIAM2S blockage significantly revoked RR-increased cervical cancer cell migration. (**A**) Representative pictures show new DNA replication in wild-type (WT) and radioresistant (RR) CaSki cells. In brief, serum-starved cells were cultured for 24 h, and then 5′-bromo-2′-deoxyuridine (BrdU; 100 µg/mL) was added to culture media 6 h before cells were fixed for BrdU staining. BrdU-positive cells (newly proliferating cells, red fluorescence) were stained using a cell proliferation assay kit, as described in Section 2.7, to estimate cell proliferation. DAPI (4′,6-diamidino-2-phenylindole) staining was shown by blue fluorescence for nuclear counterstaining. Nine to twelve randomly selected fields were inspected for counting BrdU-positive cells, with a summary shown in (**B**). For each experiment, at least 500 cells were counted to quantify BrdU-positive cells. (**C**) A summary of BrdU-positive cells designated WT and RR cervical cancer cells, which were transfected with various dosages of pcDNA3.1-Myc-His A-TIAM2S or vector alone, and *siRNA of scrambled NC* or *siRNA of TIAM2S* for 72 h, respectively. (**D**) We elucidated the in vitro impact of TIAM2S in regulating cell proliferation with or without 6 Gy radiation treatment. CaSki-WT or C33A-WT cells were pre-transfected with pcDNA3.1-Myc-His A-TIAM2S construct (3 μg) or vector alone (3 μg), respectively, whereas RR cells were pre-transfected with *siRNA of scrambled NC* (30 nM) or *siRNA of TIAM2S* (30 nM), respectively. After 72 h of transfection, designed cells were resubjected to 0 Gy or 6 Gy radiation treatment; BrdU-positive cells was assessed and a summary was provided. Data are shown as mean ± SD of three independent experiments using different batches of cells. * *p* < 0.05, ** *p* < 0.01, *** *p* < 0.001; “ns” indicates no significance (*p* > 0.05).

Hence, radiation treatment failure in cervical carcinoma is often associated with uncontrolled cell growth/propagation. We then investigated whether TIAM2S was involved in regulating RR-associated cell propagation. Replicating cells undergo DNA synthesis in a highly regulated S-phase of the cell cycle; synthetic BrdU (5-bromo-2-deoxyuridine) is a thymidine analog able to integrate into the cells of the DNA synthetic phase. Thus, to estimate new cell proliferation, the BrdU cell proliferation assay—a non-isotopic immunoassay for the in vitro quantification of BrdU incorporation into the newly synthesized DNA of actively proliferating cells—was executed, as described in Section 2.7. Briefly, serum-starved cells were cultivated and, six hours before harvest, BrdU (100 µg/mL) was added into the culture media; then, cells were fixed and stained with anti-BrdU antibody. BrdU-positive cells (newly active proliferating cells) were identified with red fluorescence and quantified by flow cytometry. DAPI (4′,6-diamidino-2-phenylindole) staining showed blue fluorescence for nuclear counterstaining. As shown in Figure 4A, a higher basal level of the BrdU-positive cell ratio is shown in CaSki-RR cells than in CaSki-WT cells. The Myc-His A-tagged human TIAM2S construct was transfected into CaSki-WT cells and incubated for 72 h, and the result showed that TIAM2S overexpression significantly enhanced the BrdU-positive cell population in CaSki-WT with a dose-dependent relationship (Figure 4A,B). In contrast, 30nM of *siRNA of TIAM2S* was transfected into CaSki-RR cells for 72 h, and TIAM2S knockdown exhibited a dose-dependent response in abolishing the BrdU-positive cell population compared to the CaSki-RR group transfected with *siRNA of scramble control* (Figure 4A,B). Hence, we further elucidated the impact of TIAM2S on CaSki-WT and C33A-RR cell propagation with or without 6 Gy radiation treatment status. WT cells were transfected with TIAM2S construct or vector control for 72 h, before they were then resubjected to 6 Gy radiation administration. After 48 h following 6 Gy radiation treatment, we found that 6 Gy radiation remarkedly reduced the basal level of cell proliferation of WT cells compared to the group without radiation treatment, and the forced introduction of TIAM2S successfully reinforced cell proliferation (Figure 4C). An amplified basal-level cell proliferation rate was detected in CaSki-RR and C33A-RR cells compared to their WT cells. TIAM2S abrogation significantly diminished CaSki-RR and C33A-RR cell proliferation in both 0 Gy and 6 Gy radiation treatment groups (Figure 4C). A similar response was revealed in a parallel experiment with C33A cervical cancer cells (Figure 4B,D). These data suggest that TIAM2S may act as a mitogen, contributing to activating the new DNA synthesis of actively proliferating cells in CaSki-RR and C33A-RR cells. Interrupting TIAM2S expression may effectively block cell growth/propagation while treating radioresistant cervical cancer cells.

### 3.5. TIAM2S Accelerated the Cell Migrative Properties of CaSki and C33A Radioresistant Cells, as Confirmed by Time-Lapse Recording Cell Movement Assay

**Figure 5:**
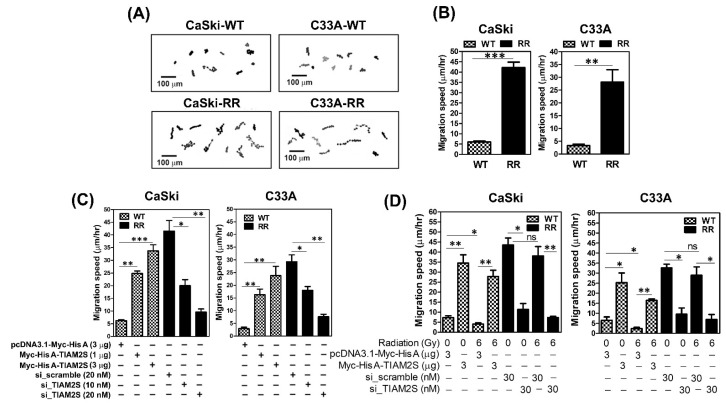
Radioresistant CaSki and C33A cells exhibited increased cell migration properties in vitro, and TIAM2S suppression remarkedly abrogated RR-enhanced cell migration. (**A**) Representative cell migration trace from a time-lapse recording of cell migration over 8 h. Scale bar = 100 μm. The results of each cell were repeated at least three times, and the results were similar. (**B**) Summary of migration speed from a time-lapse recording of cell migration over 8 h. Data are shown as mean ± SD of three independent experiments. All radioresistant C33A and CaSki cell clones underwent stable selection for 6 months after irradiation, and >20 cells per individual sample were counted. (**C**) Representative cell migration trace from a time-lapse recording of cell migration over 8 h. Designated wild-type and radioresistant CaSki and C33A cells clones were pre-transfected with various dosages of pcDNA3.1-Myc-His A-TIAM2S or vector alone, and *siRNA of scrambled NC* or *siRNA of TIAM2S*, respectively. After 72 h of transfection, cells were collected and migration was assessed, as described in Section 2.8, and summary of migration speed was shown. (**D**) We investigated the in vitro impact of TIAM2S’s contribution to modulating cell migration with or without 6 Gy radiation treatment. CaSki-WT or C33A-WT cells were pre-transfected with pcDNA3.1-Myc-His A-TIAM2S construct or vector, respectively, whereas RR cells were pre-transfected with *siRNA of scrambled NC* (30 nM) or *siRNA of TIAM2S* (30 nM), respectively. After 72 h of transfection, designed cells were resubjected to 0 Gy or 6 Gy radiation treatment, cell migration speed was assessed and summary was shown. * *p* < 0.05, ** *p* < 0.01, *** *p* < 0.001; “ns” indicates no significance (*p* > 0.05).

Radioresistance-increased cell metastasis is still the major challenge following radiation treatment failure in cervical cancer [4,5,6]. Therefore, herein, we firstly intended to clarify whether higher cell metastatic capacities were shown in radioresistant CaSki and C33A cell clones than those of parental WT cells. To measure the cell migrative capacity, the time-lapse recording cell movement assay was conducted hourly to record migrating cell paths in parental wild-type and radioresistant CaSki and C33A cells, as described in Section 2.8. We found that radioresistant CaSki and C33A cells exhibited consistently higher migration speeds than those in their parental WT cells (Figure 5A,B). Next, because we have illustrated that, in both CaSki and C33A cells, RR cell clones expressed higher TIAM2S levels (Figure 1A) than parental WT cell clones, CaSki-RR and C33A-RR cells were transfected with *siRNA of TIAM2S* for 72 h to reduce the levels. TIAM2S suppression dose-dependently abridged the elevated cell migration of the CaSki-RR and C33A-RR cells, whereas the overexpression of TIAM2S in WT cells constantly increased it (Figure 5C). Hence, TIAM2S was ectopically introduced to CaSki-WT or C33A-WT cells for 72 h, which were then resubjected to 6 Gy radiation treatment. After 48 h of radiation administration, the cell migration speed was reduced and TIAM2S overexpression accelerated CaSki-WT and C33A-WT cell migration (Figure 5D). An augmented cell migration speed was shown in CaSki-RR and C33A-RR cells compared to their WT cells, and TIAM2S blockage notably declined cell migration. There was consistently no significant difference shown between the 0 Gy and 6 Gy radiation treatment groups of radioresistant cell clones. In summary, this evidence suggests that TIAM2S may act as a pro-migration-relevant factor in CaSki-RR and C33A-RR cells, and TIAM2S blockage exerts advantageous effects in suppressing CaSki-RR and C33A-RR cell migration; this is true in both a steady state (without radiation treatment) and when radioresistant cell clones were combined with 6 Gy radiation re-challenge.

### 3.6. Escalated Lung Locomotion After Radioresistant Cells’ Injection; TIAM2S Abrogation Noticeably Interrupted Lung Locomotion in BALB/c Nude Mice

**Figure 6:**
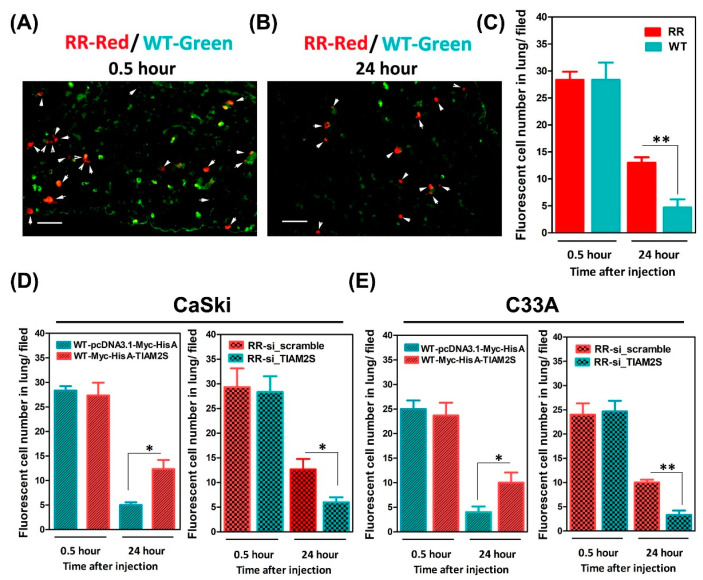
Increased lung localization and survival rates were shown in *BALB/c* nude mice after tail-vein injection of radioresistant CaSki cells; ectopic introduction of TIAM2S notably abridged radioresistance-augmented lung localization. CaSki-WT (green fluorescence) and CaSki-RR cells (red fluorescence) were mixed in a 1:1 ratio and injected into *BALB/c* nude mice via tail-vein injection, as described in Section 2.9 and Section 2.10. Representative fluorescence micrographs of frozen sections of mouse lung at (**A**) 0.5 and (**B**) 24 h after tail-vein injection are shown. White arrows indicate red fluorescent CaSki-RR cells. Scale bar = 200 μm. Similar results were collected for the six mice in each group. (**C**) Summary of number of fluorescent cells in lung (red fluorescence indicating CaSki-RR cells vs. green indicating CaSki-WT cells). Summary of number of fluorescent (**D**) CaSki or (**E**) C33A cells retained in lung. Data are mean ± SD from six independent experiments. * *p* < 0.05, ** *p* < 0.01.

Next, we evaluated whether radioresistance-elevated cell migration might increase in vivo lung metastasis. An in vivo short-term lung locomotion metastasis animal model was used, as in previous studies [15,20,21]. In brief, wild-type (WT) and radioresistant CaSki cells were mixed and injected through the tail vein into *BALB/c* nude mice. Cells injected in the tail vein would flow through to the heart and then into the lung circulatory system. The lungs are the first microvascular area that cells will flow through, and injected cells can be retained and detected in the endothelial cell lining of microvessels. To track the injected cells, CaSki cells were pre-labeled with fluorescent living dyes prior to the tail-vein injection. CaSki-WT cells were labeled with green fluorescent dye and CaSki-RR cells with red fluorescent dye, as described in Section 2.9. Then, the labeled cells were mixed in a 1:1 ratio and injected into mice. Later, mice were sacrificed at 0.5 or 24 h after tail-vein injection, and the lungs were harvested for acquiring frozen sections and specimen staining, as described in Section 2.10. After 0.5 h of tail-vein injection, the ratio of CaSki-RR (red fluorescence) to CaSki-WT cells (green fluorescence) retained in the lung was nearly 1:1 (after normalizing to the composition of the suspension before injection) (Figure 6A,C). These data showed that the injected cells (mixed CaSki-WT and CaSki-RR cell suspension) also arrived at the lungs in the very early initiation stage (Figure 6A,C). The phenomenon observed was identical to that reported in the literature [20,21]. After 24 h of injection, there was a significantly elevated number of CaSki-RR cells retained in lungs compared to in mice that received a CaSki-WT cell injection (Figure 6B,C). Because we observed that TIAM2S exhibited pro-cell migrative properties in radioresistant cells (Figure 5C,D), we next decided to observe whether RR cells with higher invasive/migrative properties would have a better chance of further migration into the endothelium of lung microvessels and retention in the lungs. Thus, in another parallel experimental set, CaSki-WT cells were pre-transfected with Myc-His A-tagged TIAM2S or control vector for 72 h, respectively. Later, prior to the tail-vein injection in nude mice, the WT-Myc-His A-tagged TIAM2S cell group was labeled with red fluorescence and WT-pcDNA3.1-Myc-His A vector control cells with green. As shown in Figure 6D, the fluorescent cell number ratio of the WT-Myc-His A-tagged TIAM2S (red) group and WT-vector control (green) group retained in the lungs was nearly 1:1 after 0.5 h. After 24 h, a more elevated cell number was shown in the mice that received WT-Myc-His A-tagged TIAM2S compared those that received the WT-vector control cells (Figure 6D). Additionally, CaSki-RR cells were transfected with *siRNA of TIAM2S* (RR-si_TIAM2S) or *siRNA of scramble NC* (RR-si_scramble) for 72 h, respectively. Prior to tail-vein injection, RR-scramble cells were then labeled with red fluorescence dye, whereas RR- si_TIAM2S cells were labeled with green. In the same way, the fluorescent cell number ratio of the RR-scramble group (red) and RR- si_TIAM2S group (green) in lungs was also nearly 1:1 after 0.5 h, whereas the cell number of the RR-si_TIAM2S group in lungs was dramatically reduced compared to those in the RR-scramble group after 24 h (Figure 6D). A similar phenomenon was detected in a parallel experiment set of C33A cells (Figure 6E). Our data suggest that the aberrant increased TIAM2S level in radioresistant cervical cancer cells and the TIAM2S-mediated pro-cell migrative property (Figure 5C,D) may promote the advancement of in vivo lung metastasis. Impeding TIAM2S expression may help to alleviate radioresistance-enhanced lung metastasis.

## 4. Discussion

The tolerance of cervical cancer to radiotherapy has become a major treatment complication. Radiation treatment failure is often accompanied by a loss of radiosensitivity, apoptotic resistance, uncontrolled cell propagation, and metastasis [22,23,24]. Nevertheless, its detailed mechanism remains uncertain. Interestingly, via bioinformatics analysis, we identified a unique feature of aberrant amplified TIAM2S in the group of the cervical cancer patients with intrinsic radioresistance after 6 months of radiotherapy, in comparison to those who saw a complete response. Currently, there is little available detailed information on the biological functions of TIAM2S in human disease. We are thus interested in further elucidating the impact of TIAM2S participation in regulating radiation resistance in cervical cancer. The TIAM2S profiling pattern observed among our established WT and RR cervical cancer clones was in line with bioinformatics evidence. Herein, we reported a consistent increase in TIAM2S levels among the three established radioresistant (RR) cells compared to the parental wild-type (WT) group. A radiation survival curve was executed on cells in log-phase growth via a clonogenic comet assay. We then revealed that the overexpression of TIAM2S in WT cervical cancer cells significantly enhanced the surviving fraction (and decreased the radiosensitivity), whereas TIAM2S suppression decreased the surviving populations under 6 Gy radiation treatment. TIAM2S blockage in radioresistant CaSki and C33A cells reinforced cell apoptosis, while the ectopic expression of TIAM2S reduced WT cell apoptosis. The ectopic introduction of TIAM2S into the WT cell group markedly stimulated new DNA synthesis/cell proliferation according to a BrdU incorporation cell proliferation assay, whereas TIAM2S knockdown in the RR group effectively diminished cell proliferation. Hence, by using a time-lapse recording assay to measure the cell movements, we discovered that TIAM2S blockage dramatically attenuated the cell migration speed of the RR group, while TIAM2S overexpression remarkably enhanced WT cell mobilities. Notably, by using the in vivo short-term lung locomotion metastasis animal model, we revealed increased lung localization in *BALB/c* nude mice that received the CaSki-RR cell injection compared to those that received the CaSki-WT cells. TIAM2S suppression reduced radioresistance-increased lung locomotion. In sum, our study provides evidence suggesting TIAM2S might operate as an innovative signature in cervical cancer resistant to radiotherapy. TIAM2S displayed pluripotent roles in regulating the multiple steps of cervical cancer’s complex tolerance to radiotherapy, including anti-radioresistance, anti-apoptosis, induced cell proliferation, and anti-cell migration/metastasis. TIAM2S blockage may drive multiple advantages for enhancing radiosensitivity during radiotherapy, triggering cell apoptosis, restricting uncontrol cell propagation and preventing subsequent radioresistance-enhanced metastasis during radiation treatment for cervical cancer.

Radiotherapy is an effective treatment option for cancer patients, and the development of tumor radiation resistance is a complex process encompassing various factors and mechanisms [25,26,27,28,29,30]. During radiotherapy, radiation is able to directly attack biologically active macromolecules, such as DNA and enzymes, causing aberrations in their structure and function. Radiation can also cause the ionization and excitation of water molecules, induce the production of reactive oxygen species and cause oxidative stress (OS), leading to a complex response including cell cycle arrest in G1 and G2, apoptosis, and DNA repair; the remaining viable cells form radioresistant survivors [26,27,28]. Regarding radioresistance in cervical cancer, some progress been reported in recent decades. Y. Zhao et al. reported that Metadherin (MTDH) may participate in modulating radiation sensitivity. MTDH is also known as AEG-1 (Astrocyte Elevated Gene 1); elevated levels have been reported in many cancers, including breast, prostate, liver, and esophageal cancers, whereas its expression is low or absent in non-malignant tissues. Zhao et al. then demonstrated that MTDH gene knockdown reduces the expression of Ku70 and Bcl2, which affects double-strand break (DSB) repair by damaging DNA nonhomologous end joining (NHEJ) and improving radiation sensitivity [31]. Zhou S et al. identified a serine protease kallikrein-related peptidase 5 (KLK5), which is widely expressed in the epidermis, is a vital modulator of skin homeostasis, and may be involved in regulating cervical cancer radioresistance. They further demonstrated that KLK5 inhibition in cervical cancer cells decreases radioresistance by downregulating G2/mitotic-specific cyclin-B1 (cyclin B1) expression and blocking the transition to the G2/M phase [32]. Vassilev LT et al. reported that radiation-induced ataxia–telangiectasia mutated (ATM) and its target p53, which is a well-known modulator that enhances radioresistance in various cancers, may act as possible biomarkers of cervical cancer cell response to radiation treatment [33]. They also further demonstrated that employing an ATM kinase inhibitor may be an effective radiosensitizer and adjuvant therapy for patients. For example, K.M. et al. identified multiple DNA damage-repair genes associated with a distinct prognosis for cervical cancer following postoperative radiation [34]. They reported that TP53BP1 (p53-binding protein 1), MCM9 (minichromosome maintenance 9, at higher than mean levels), and SIRT6 (sirtuin 6, at lower than mean levels) were linked with an increased cervical cancer recurrence/progression. However, the detailed mechanisms responsible for the development of radioresistance in cervical cancer are yet to be determined. Experts are seeking potential treatment targets or potent prognosis biosignatures for radioresistance, as improving survival rates for cervical cancer patients is an urgent need. In this study, we revealed greater aberrant TIAM2S augmentation in radioresistant HeLa, CaSki, and C33A cells compared to those from their parental wild-type (WT) cells (Figure 1A,B). We further demonstrated that TIAM2S knockdown in CaSki-RR and C33A-RR cells reduced clonogenic survival rates, while its forced introduction in parental CaSki-WT and C33A-WT cells enhanced survival (Figure 2A,B). Our data provide imperative evidence suggesting that TIAM2S may display an innovative radio-protective role for cervical cancer cells against radiation damage; it may also reduce radiosensitivity and may result in increased cell survival rates alongside strengthened radioresistant properties.

Hence, cancer cells’ impeded responsiveness to radiation treatment and subsequent aberrant cell propagation in radioresistant cancers remains a major challenge in cervical cancer treatment. Hu Y et al. reported that hepatocyte nuclear factor 1A (HNF1A) transcription factor functions as a protooncogene in cervical cancer and actively partakes in the proliferation of cervical cancer cells. HNF1A overexpression significantly enhances the radiation resistance of cervical cancer cells both in vitro and in vivo [35]. TieXu et al. reported that silencing NIMA Related Kinase 2 (NEK2) significantly attenuates the Wnt/β-catenin signaling pathway, resulting in impaired cell growth and proliferation and increased radiosensitivity. NEK2 upregulation promotes radiation resistance in cervical cancer [36]. Samarzija I et al. demonstrated that the Sonic Hedgehog (Shh) ligand induces cervical cancer cell proliferation, while Hh signaling inhibition decreased proliferation and stimulates the apoptosis [37]. Herein, we detected augmented basal-cell proliferation rates in CaSki-RR and C33A-RR cells compared to their parental WT cells. The ectopic introduction of TIAM2S into CaSki-WT and C33A-WT cells significantly induced cell propagation and reduced apoptosis, whereas TIAM2S knockdown blocked CaSki-RR and C33A-RR cell propagation and increased apoptosis (Figure 3 and Figure 4). We also further revealed that TIAM2S abrogation significantly abolished radioresistant CaSki and C33A cell proliferation under 6 Gy radiation treatment (Figure 4C,D), implying that employing a TIAM2S inhibition strategy when treating radioresistant cervical cancer cells may mitigate uncontrolled cell growth/propagation. These data suggest that TIAM2S may act as a mitogen and help stimulate radioresistant CaSki and C33A cervical cancer cell proliferation. Meanwhile, TIAM2S may also manifest as an anti-apoptosis factor, diminishing radioresistant cell apoptosis. Collectively, our data provide vital evidence showing that TIAM2S interruption may trigger dual beneficial effects, reinforcing cell apoptosis, restricting newly synthesized DNA, and thus disrupting cell propagation and the development of radioresistance.

Furthermore, for cervical cancer patients, radiation therapy failure is often accompanied by metastasis [23,24]. Thus, we then explored whether TIAM2S impacted radioresistance-increased metastasis using an in vitro cellular model and in vivo animal model. Our data revealed that CaSki-RR and C33A-RR cells increased cell migrative speeds more than the WT counterparts, as confirmed by time-lapse recording assay. TIAM2S overexpression increased parental wild-type CaSki and C33A cell migration, whereas its blockage in radioresistant cells reduced cell mobilities (Figure 5). We then further delineated the in vivo influence of TIAM2S on metastasis. Noticeable higher radioresistant CaSki and C33A cell populations were retained in the lung 24 h after injection compared to those in the parental WT cell group (Figure 6C). TIAM2S suppression dramatically diminished the CaSki-RR and C33A-RR cells in the lung, whereas its exogenous introduction successfully retained them (Figure 6D,E). Interestingly, several previous studies have shown that TIAM2S is only detected in the peripheral tissue/organ when in the malignant stage. According to Yen, W.H. et al., in the vast majority (around 86%) of hepatocellular carcinoma (HCC) specimens, TIAM2S overexpression and exogenously introduced human TIAM2S protein provoke liver cancer tumorigenesis and metastasis [18,38]. Later, TIAM2S was reported to enhance non-small cell lung cancer (NSCLC) cell invasion and motility [39]. These data are consistent with our findings. Additionally, TIAM2S is most known for its role in regulating microglia neuroinflammation [13] and aggravated age-related spatial memory impairment [14]. Our collaborative team recently demonstrated that TIAM2S aggravates proinflammatory immune microenvironments tolerant to enhanced colorectal carcinogenesis [40], implying that aberrant TIAM2S expression likely contributes to tumor malignancies. In this study, we provided new insights, demonstrating that radioresistance-enhanced cell migration may provoke the in vivo lung metastasis in cervical cancer. Exogenously introduced TIAM2S might play a pro-metastasis role, impeding TIAM2S and effectively mitigating radioresistance-enhanced lung metastasis.

## 5. Conclusions

Radioresistance remains a major clinical obstacle in cervical cancer treatment. The details of its mechanism of action remain largely uncertain. Herein, we reported an additional piece of evidence for this problem. We demonstrated that TIAM2S may serve as a specific feature in assessing radiosensitivity and cervical cancer prognosis. Targeting TIAM2S may be an innovative therapeutic strategy in sensitizing cervical cancer after radiotherapy, triggering the apoptotic response, and blocking cell propagation. Furthermore, we demonstrated that TIAM2S suppression may represent an informed strategy for interrupting radioresistance-enhanced cell migration or consequent lung metastasis. To the best of our knowledge, all the findings presented here are innovative and novel. Taken together, this study provides multiple fresh insights; during radiotherapy, a combined impaired TIAM2S therapeutic strategy might intensify the responsiveness to radiation, stimulate cell apoptosis, and suppress migration/metastasis, thus aiding in the treatment of radioresistant cervical cancer.

## Data Availability

The original contributions presented in this study are included in the article/Supplementary Material. Further inquiries can be directed to the corresponding authors.

## Supplementary Materials

The following supporting information can be downloaded at: https://www.mdpi.com/article/10.3390/cells14050339/s1, Figure S1: Bioinformatics analysis from the Gene Expression Omnibus (GEO) database Accession Viewer; Figure S2: Expression of TIAM2S protein in CaSki and C33A cells; Table S1: Plating efficiency (%) of clonogenic assay.
