# Supplementary material for: TIAM2S Operates Multifaced Talents to Alleviate Radiosensitivity, Restrict Apoptosis, Provoke Cell Propagation, and Escalate Cell Migration for Aggravating Radioresistance-Intensified Cervical Cancer Progression
Source: Cells. 2025 Feb 26;14(5):339. doi: 10.3390/cells14050339 (PMC11898548; doi:10.3390/cells14050339)
Supplement: Supplementary file 1 [file cells-14-00339-s001.zip › (0214 Final ) Supplementary Figures and Legends (cells_TIAM2S and RR).pdf]

Supplementary Figures and Legends

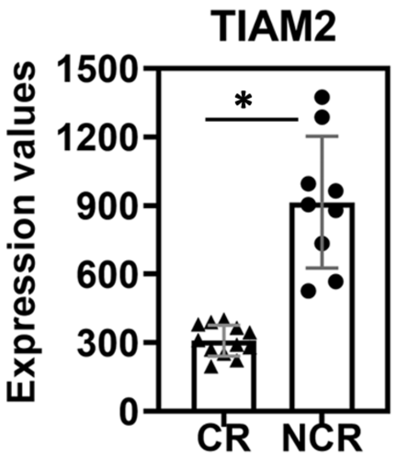

Figure S1. Bioinformatics analysis from the Gene Expression Omnibus (GEO) database Accession Viewer, supported by the National Center for Biotechnology Information (NCBI) at the National Library of Medicine (NLM) https://www.ncbi.nlm.nih.gov/geo/ (Gene expression datasets Accession Number: GSE56363), identified an amplified TIAM2 transcript level in cervical cancer samples with 6-month non-complete response (defined as "NCR" group; 9 patients) compared with complete response (defined as "CR" group; 12 patients) after the external beam radiotherapy (EBRT). Briefly, 21 patients with locally advanced squamous cell carcinoma (FIGO stage IIB-IIIB) were enrolled in the genomics study. A tissue fragment from a primary biopsy specimen was harvested from each patient prior to the therapy. Tissue samples were stored in liquid nitrogen until use for RNA extraction. Primary biopsies were divided according to the patient's 6 months clinical response after radiotherapy. One-color microarray experiment was performed analyzed for whole human gene expression to measure differences in gene expression between cervical cancer samples with 6-month complete response (N=12) and non-complete response (partial response and stable disease; N=9). Complete response group was considered as reference. Integrated Density Value from microarray was shown as "Expression value"; *$P<0.05$, for the CR" group versus NCR" group.

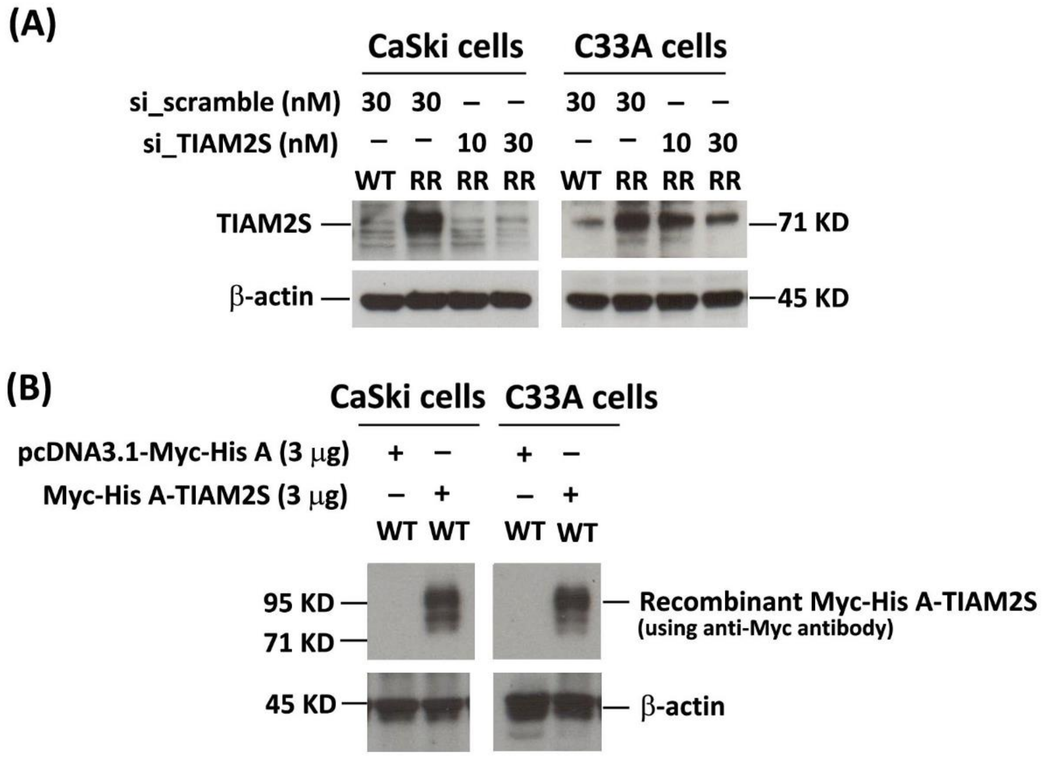

Figure S2. Expression of TIAM2S protein in CaSki and C33A cells. **(A)** Radioresistant CaSki and C33A cells (with higher TIAM2S expression) were transfected with the scramble siRNA (a negative control for siRNA) or transfected with the siRNA of TIAM2S for 72 hours, respectively. In contrast, parental CaSki-WT and C33A cells (with lower or little TIAM2S expression) were transfected with the scramble siRNA for 72 hours. Expression of endogenous TIAM2S protein (71 KD) was determined by Western Blot. **(B)** Parental CaSki-WT and C33A-WT cells were transfected with the pcDNA3.1-Myc-His A vector alone or transfected with pcDNA3.1-Myc-His A-TIAM2S construct for 72 hours, respectively. Ectopic overexpression of recombinant Myc-His A-TIAM2S (95 KD) was recognized by anti-Myc tag antibodies, and β-actin (45 KD) served as a loading control.
