# Supplementary material for: TIAM2S Operates Multifaced Talents to Alleviate Radiosensitivity, Restrict Apoptosis, Provoke Cell Propagation, and Escalate Cell Migration for Aggravating Radioresistance-Intensified Cervical Cancer Progression
Source: Cells. 2025 Feb 26;14(5):339. doi: 10.3390/cells14050339 (PMC11898548; doi:10.3390/cells14050339)
Supplement: Supplementary file 1 [file cells-14-00339-s001.zip › Table S1.pdf]

Table S1

Table S1 Plating efficiency (%) of clonogenic assay

**Cells and condition**	**Colony number of non-irradiated cells**
CaSki RR/si_scr	58.6 ± 1.8
CaSki RR/si_TIAM2S	50.3 ± 0.9*
CaSki WT/pcDNA3.1-Myc-His A vector control	48.9 ± 0.6
CaSki WT/pcDNA3.1-Myc-His A-TIAM2S	56.7 ± 2.1*
C33A RR/si_scr	77.1 ± 0.5
C33A RR/si_TIAM2S	71.5 ± 1.4*
C33A WT/pcDNA3.1-Myc-His A vector control	70.3 ± 0.2
C33A WT/pcDNA3.1-Myc-His A-TIAM2S	80.6 + 0.7*

Abbreviations: WT, wild-type; RR, radioresistance; si_scr, small interfering scrambled control RNA; si_TIAM2S, small interfering T-cell lymphoma invasion and metastasis 2; *$P<0.05$* versus control group.
